# Supplementary material for: A Comprehensive Study of the Microbiome, Resistome, and Physical and Chemical Characteristics of Chicken Waste from Intensive Farms
Source: Biomolecules. 2022 Aug 17;12(8):1132. doi: 10.3390/biom12081132 (PMC9406075; doi:10.3390/biom12081132)
Supplement: Supplementary file 1 [file biomolecules-12-01132-s001.zip › Table S1-S2.pdf]

Table S1. Antimicrobial susceptibility discs used for the isolated strains' phenotypic characterization

Selective medium	Antibiotics (abbreviation, concentration)
Simmons Citrate Agar	IMP, 10 µg; CIP, 5 µg; CTX, 5 µg; CN, 10 µg
Eosin Methylene Blue Agar	IMP (10 µg), CIP (5 µg), CTX (5 µg), CN (10 µg)
MacConkey Agar	IMP (10 µg), CIP (5 µg), CTX (5 µg), CN (10 µg)
Brilliance VRE agar	IMP (10 µg), CIP (5 µg), VAN (30 µg), QD (15 µg)
Cetrimide Agar	IMP (10 µg), CIP (5 µg), CTX (5 µg), FEP (30 µg)
Mannitol Salt Agar	OX (1 µg), TE (30 µg), VAN (30 µg), ER (15 µg)

IMP - imipenem, **CIP** – ciprofloxacin, **CTX** – cefotaxime, **CN** – gentamicin, **VAN** – vancomycin, **QD** - quinupristin/dalfopristin, **FEP** – cefepime, **OX** – oxacillin, **TE** – tetracycline, **ER** - erythromycin

Table S2. Antibiotic resistance [%] of isolated strains form two types of chicken wastes Chicken Litter (CL) and Chicken Manure (CM)

Antibiotic (n=total number of strains tested)	Sensitive strains (%)	Intermediate resistant strains (%)	Resistant strains (%)
Amoxicillin/Clavulanic acid (n=57)	17 (29.8%)	4(7%)	36(63.2%)
Piperacillin/Tazobactam (n=72)	43 (59.7%)	17 (23.6%)	12 (16.7%)
Cefuroxime (n=57)	32 (56.1%)	4 (7.1%)	21 (36.8%)
Ceftazidime (n=57)	26 (45.6%)	1 (1.8%)	30 (52.6%)
Imipenem (n=72)	42(58.3%)	25 (34.7%)	5 (7%)
Meropenem (n=72)	68 (94.4%)	2 (2.8%)	2 (2.8%)
Gentamicin (n=91)	45 (49.5%)	-	46 (50.5%)
Amikacin (n=72)	58 (80.5%)	1 (1.4%)	13 (18.1%)
Tobramycin (n=72)	33 (45.8%)	-	39 (54.2%)
Cefotaxime (n=57)	29 (50%)	1 (1.7%)	27 (48.3%)
Ciprofloxacine (n=91)	23 (25.3%)	12 (13.2%)	56 (61.5%)
Cefepime (n=57)	54 (94.8%)	2 (3.5%)	1 (1.7%)
Cefuroxime-axetil (n=57)	26 (45.6%)	-	31 (54.4%)
Tigecycline (n=72)	45 (62.3%)	1 (1.3%)	26 (36.4%)
Trimetoprim/Sulfametoxazol (n=90)	65 (72.2%)	2 (2.2%)	23 (25.6%)
Levofloxacin (n=34)	5 (14.7%)	4 (11.8%)	25 (73.5%)
Erythromycin (n=19)*	8 (42.1%)	-	11 (57.9%)
Tetracycline (n=19)*	-	-	19 (100%)
Clindamycin (n=19)*	2 (10.5%)	2 (10.5%)	15 (79%)

*only *Staphylococcus luteus* strains
