# Supplementary figures and images for: A Comprehensive Study of the Microbiome, Resistome, and Physical and Chemical Characteristics of Chicken Waste from Intensive Farms
Source: Biomolecules. 2022 Aug 17;12(8):1132. doi: 10.3390/biom12081132 (PMC9406075; doi:10.3390/biom12081132)

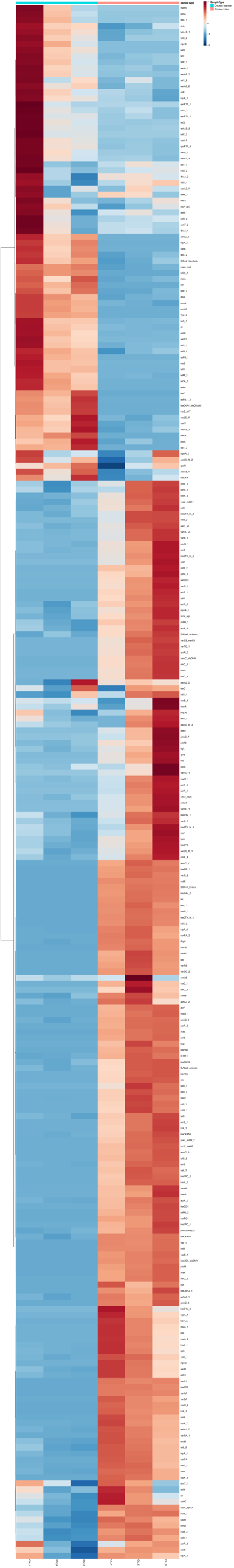

Supplement: Supplementary file 1 [file biomolecules-12-01132-s001.zip › Figure S1 ARG in chicken waste (heatmap).pdf]
